# Gut Microbiome Changes in Patients With Idiopathic Normal Pressure Hydrocephalus

**DOI:** 10.1097/WAD.0000000000000613

**Published:** 2024-04-11

**Authors:** Emilia Brandt, Anne Koivisto, Pedro Pereira, Ella Mustanoja, Petri Auvinen, Toni Saari, Juha-Matti Lehtola, Sanna Hannonen, Minna Rusanen, Ville Leinonen, Filip Scheperjans, Virve Kärkkäinen

**Affiliations:** Departments of * Neurology; †† Neurosurgery, Institute of Clinical Medicine, School of Medicine, University of Eastern Finland; † NeuroCenter, Kuopio University Hospital, Kuopio; ‡ Department of Neurosciences, Faculty of Medicine; ¶ Department of Geriatrics, Helsinki University Hospital Helsinki; # Institute of Biotechnology, University of Helsinki, Helsinki Institute of Life Sciences; ‡‡ Clinicum, University of Helsinki; Departments of § Geriatrics; ∥ Neurology, Helsinki University Hospital, Helsinki; ** Department of Psychiatry, Turku University Hospital, Turku, Finland

**Keywords:** hydrocephalus, normal pressure, neurodegenerative diseases, iNPH, cognition disorders, biomarkers, gastrointestinal microbiome, microbiota, humans

## Abstract

**Background:**

The gut microbiome is a complex system within the human gastrointestinal tract. The bacteria play a significant role in human health, and some can promote inflammation and pathologic processes through chemical interactions or metabolites. Gut microbiome dysbiosis has been linked to some neurological and other diseases. Here we aimed to examine microbiome differences between patients with a progressive neurological disorder, idiopathic normal pressure hydrocephalus (iNPH), compared with healthy controls (CO).

**Methods:**

We recruited 37 neurologically healthy CO and 10 patients with shunted iNPH. We evaluated these participants’ cognition using the CERAD-NB test battery and CDR test, and collected a variety of information, including about dietary habits and health. We also collected fecal samples, which were subjected to 16S amplicon sequencing to analyze differences in gut microbiome composition.

**Results:**

We found that the iNPH group exhibited significantly different abundances of 10 bacterial genera compared with the CO group. The *Escherichia/Shigella* and *Anaeromassilibacillus* genera were most remarkably increased. Other increased genera were *Butyrivibrio*, *Duncaniella*, and an unidentified genus. The decreased genera were *Agathobaculum*, *Paramuribaculum*, *Catenibacterium*, and 2 unidentified genera.

**Conclusions:**

Here we report the first identified microbiome differences in iNPH patients compared with healthy controls.

Idiopathic normal pressure hydrocephalus (iNPH) is a progressive neurological disease that manifests as a cerebrospinal fluid circulation disorder, causing symptoms such as cognitive impairment, urinary dysfunction, and gait disturbance.^1,2^ It can be challenging to differentiate iNPH from other neurodegenerative disorders, such as Alzheimer disease (AD), since there are no iNPH-specific hallmarks if the patient also exhibits neuropathologic findings similar to AD.^3^ Notably, it is important to diagnose iNPH as early as possible because it can be treated with shunt surgery, which improves cognitive function and alleviates other typical iNPH symptoms in most cases.^4^ Due to the progressive nature and potential treatability of iNPH, there is an urgent need to identify new accessible and cost-effective biomarkers for neurodegenerative disorders, both for clinical and differential diagnostics.

The gastrointestinal system and the brain are connected by multiple neural and chemical pathways, forming a complex bidirectional gut-brain axis. Growing evidence supports a connection between the gut microbiome and various neurological diseases, making the gut microbiome an attractive potential source of biomarkers for early diagnosis.^5–8^ The literature includes reports of altered microbiome composition in Parkinson disease and AD.^6,9,10^ In the present study, we aimed to investigate the gut microbiome in iNPH patients and examine potential differences from healthy controls. To our knowledge, this topic has not previously been studied.

## METHODS

### Ethics Statement

This study was evaluated and approved by the Ethical Committee of Kuopio University Hospital (Dnro: 482/2017 and 276/2016) and adhered to the principles of the Declaration of Helsinki. Before participation, all study participants read the information letter and signed an informed consent.

### Study Design, Participants, and Protocol

The study population comprised a total of 47 voluntary participants. The iNPH group included 10 individuals who had been diagnosed with idiopathic normal pressure hydrocephalus (iNPH group) and who did not have any other neurodegenerative disorders. The iNPH group was formed using the NPH registry in Kuopio, which included 763 iNPH patients (http://www.uef.fi/nph). The control group (CO) included 37 cognitively healthy individuals who were recruited by the Brain Research Unit of the University of Eastern Finland.

The iNPH diagnoses were clinically evaluated by a neurosurgeon, including with an MRI brain scan.^1,2^ The iNPH participants had all undergone shunt surgery, during which a right frontal cortical biopsy was obtained, confirming no AD-related pathology. These participants also exhibited no other neurodegenerative disorders or brain comorbidities. The patients were in good condition, had shown responses to the shunt treatment, and presented no gait disturbances or urinary dysfunctions.

All study participants were clinically examined by a neurologist and participated in a demographic interview. Cognition was evaluated using the Consortium to Establish a Registry for Alzheimer Disease Neuropsychological Test Battery (CERAD-NB), and Clinical Dementia Rating (CDR).^11,12^ The CDR questionnaire includes questions relating to cognition and functional abilities and was used to assess daily function and cognitive decline.^13^

The participants did not exhibit any anamnestic functional or cognitive decline based on the findings of the CERAD-NB, CDR, or demographic interview. The acceptance criteria for the control group included performance within the normal limits on all cognitive tests. The exclusion criteria for both groups were diabetes, depression, or other brain disorders (eg, Parkinson disease or severe dementia).

Venous blood samples were collected to detect the apolipoprotein E (ApoE) ε4 alleles. ApoE genotyping was performed using the QIAamp DNA blood mini extraction kit (QIAGEN), as previously described by Hannonen and colleagues.^14,15^ None of the iNPH participants carried the Alzheimer disease risk allele ApoE ε4, which is not associated with iNPH; therefore, the iNPH group was successfully formed without the most common genetic risk factor for AD.^16,17^ The CO group had a higher prevalence of APOE ε4; however, the healthy control participants had no clinical signs of AD. APOE ε4 is a more common allele in Finland compared with the rest of Europe. The underlying reason is unclear, warranting further studies. Importantly, from this relatively small sample size, we were able to differentiate between people without vascular dementia or AD.

To examine potential confounding factors, we also collected a large amount of demographic data from the study population, including information about medications and different comorbidities, such as cardiovascular and metabolic diseases and gut disorders. We also recorded a large amount of information regarding dietary factors, including possible diets and the use of different food products, such as dairy or meat.

### Collection of Samples (FS)

The study subjects collected stool samples at home into collection tubes prefilled with DNA stabilizer (PSP Spin Stool DNA Plus Kit; STRATEC Molecular). These samples were stored in the freezer until transport. Once received at the Brain Research Unit at the University of Eastern Finland, the samples were stored at −80°C. Finally, the samples were transported on dry ice to Helsinki for further analysis.

### DNA Extraction and Sequencing Methods

We extracted bulk DNA from the stool samples using the PSP Spin Stool DNA Plus Kit (STRATEC Molecular). Each extraction batch included one blank sample to assess potential contamination. The V3-V4 regions of the 16S rRNA gene were amplified following a previously published protocol, with modifications as described by Aho and colleagues in 2019.^18,19^ The obtained PCR amplicon pool was checked using Fragment Analyzer (Advanced Analytical Technologies Inc., Ankeny, IA). Every PCR batch included a blank sample (with no added DNA template) to assess potential contamination. Finally, the PCR products were sequenced using Illumina MiSeq (v3 600 cycle kit), with 326 bases for the forward read and 278 bases for the reverse read. Barcodes were selected using the BARCOSEL tool.^20^

### Bioinformatics Methods and Statistical Analysis

Primer trimming of the sequences was performed using Cutadapt, v. 3.7.^21^ Quality trimming, inferring amplicon sequence variants (ASVs), and taxonomic classification were performed using DADA2, v.1.18.0, run in R v.4.0.5.^22,23^

The alpha diversity indices Observed Richness, Chao1, and ACE are indicators of the taxa richness of the microbiome, that is, how many taxa are present in each fecal sample. Richness describes the number of taxa inhabiting a certain area, whereas evenness describes the heterogeneity of taxa.^24^ Alpha diversity, which can be defined as a measure of intrasample diversity, was analyzed using data at the ASV level, with Wilcoxon rank-sum tests at an alpha cutoff of *P*≤0.05.

We used distance-based multivariate analysis methods to assess beta diversity and evaluate the effects of clinical variables on microbiome composition, and the potential need to adjust the differential abundance models for confounding effects. Beta diversity can be defined as a measure of intersample diversity, which is represented here by between-sample distances based on taxonomic composition. Data at the genus level were rarefied to an even sequencing depth per sample equivalent to the lowest sample size (34,487 sequences), which we then used to calculate the Bray-Curtis intersample distances. The resulting distance matrix was used with Permutational Multivariate Analysis of Variance (PERMANOVA), as implemented in the R package vegan. Due to the large number of clinical variables relative to the number of subjects, we initially assessed each variable’s impact in univariable (ie, single explanatory variable) PERMANOVA models. Those that were statistically significant at *P*≤0.05 were shortlisted for further analysis. The resulting shortlisted variables were checked for multicollinearity and then were tested together in a multivariable (ie, multiple explanatory variables) model to obtain the final beta-diversity (PERMANOVA) model.

Differential abundance analysis was performed using the R package DESeq2, with genus-level data, as in the beta-diversity analysis.^25^ Results were considered statistically significant if *P* was ≤0.05 after correction for multiple comparisons using the Benjamini-Hochberg method (FDR, here reported as the “adjusted *P*-value”). Size factor estimation was performed with the “poscounts” method. Effect sizes are reported as log2 fold changes after application of the “apeglm” method for shrinkage.^26^ The DESeq2 model was a univariable model with only the main grouping variable (CO/iNPH), because the PERMANOVA model suggested that it was not necessary to adjust for potential confounding effects from other clinical variables.

### Statistical Demographics Analysis

The demographic data and CERAD results were analyzed using SPSS (Version 27.0; IBM, Armonk, NY). The χ^2^ test or the Fisher exact test, as applicable, was used to analyze between-group differences in categorical variables (sex and *ApoE-*ε4 carrier). First, for continuous variables (age, education, and the CERAD-NB subtest results), the χ^2^ test was used to investigate whether the model followed an approximately normal distribution, followed by a one-way ANOVA. For post hoc comparisons of the demographic variables, we used the Bonferroni correction.

## RESULTS

### Demographics

Table 1 presents the demographics and CERAD-NB results of the study groups. We found a statistically significant between-group difference regarding the *APOE*-ε4 allele. None of the iNPH participants had *APOE*-ε4, whereas 37.8% of the healthy controls carried a single *APOE*-ε4 allele (Table 1). The CO and iNPH study groups also significantly differed on 7 CERAD-NB subtests and only showed nearly equal performances on the Boston Naming Test, Constructional Praxis, and Constructional Praxis savings. With regards to study population demographics, we analyzed multiple variables regarding diet, comorbidities, and medications and detected no significant between-group differences (data not shown).

**TABLE 1:** Study Population Demographics

	CO N=37	iNPH N=10	*P*
Age, y	71.0 (5.1)	75.9 (5.7)	0.061
Sex, female %	54 (20)	70 (7)	0.481
Education, y	12.6 (4.3)	10.0 (3.5)	0.261
ApoE allele ε4 carrier %	37.8 (14)	0	**0.022**
CERAD-NB (max score)			
Verbal fluency (animals in 60 s)	25.1 (7.3)	15.5 (5.2)	**<0.001**
Boston naming test (15)	13.4 (1.7)	12.7 (1.9)	1.000
MMSE (30)	28.4 (1.5)	24.7 (3.6)	**<0.001**
Wordlist learning (30)	23.1 (3.1)	15.9 (2.0)	**<0.001**
Wordlist savings (%)	95.1 (10.6)	56.3 (24.5)	**<0.001**
Wordlist recognition (%)	98.2 (3.6)	83.5 (21.5)	**<0.001**
Constructional praxis (11)	10.5 (1.0)	9.2 (1.8)	0.133
Constructional praxis savings (%)	95.1 (10.1)	87.8 (26.1)	1.000
Clock drawing (6)	5.5 (0.6)	4.4 (1.2)	**0.013**
Global memory score (30)	27.8 (1.8)	21.8 (2.7)	**<0.001**

Results are shown as mean (SD) or n (%).

Global memory score is a sum of wordlist delayed recall and wordlist recognition.

CO indicates control; iNPH, idiopathic normal pressure hydrocephalus.

Bolded *P*-values indicate statistically significant differences between groups (*P*≤0.05).

### Microbiome Analysis

The abundances of 10 different bacterial genera significantly differed between the groups (Table 2). Compared with the CO group, the iNPH group showed increased abundances of both *Escherichia/Shigella* and *Anaeromassilibacillus* genera, and slightly increased abundances of the *Butyrivibrio* and *Duncaniella* genera and of an unidentified genus of the Alphaproteobacteria class. In addition, the iNPH group exhibited significantly decreased abundances of the following genera: *Agathobaculum*, *Paramuribaculum*, *Catenibacterium*, an unidentified genus of the Muribaculaceae family, and an unidentified genus of the Bacteroidales order.

**TABLE 2:** Bacterial Genera That Significantly Differed in iNPH Patients Compared With Healthy Controls

Genus	iNPH vs. CO	log_2_ fold change	*P*_adj_
*Escherichia*/*Shigella*	**+**	3.65	0.0415
*Butyrivibrio*	**+**	2.48×10^−8^	9.30×10^−7^
Unknown genus 7	**+**	4.25×10^−8^	0.000329
*Anaeromassilibacillus*	**+**	3.13	0.000329
*Duncaniella*	**+**	6.58×10^−8^	0.000405
Unknown genus 3	**−**	−1.85×10^−7^	0.00411
*Agathobaculum*	**−**	−5.23×10^−6^	0.0489
*Paramuribaculum*	**−**	−2.03×10^−7^	0.0326
Unknown genus 9	**−**	−1.84×10^−7^	0.000329
*Catenibacterium*	**−**	−8.18×10^−8^	0.000180

+ indicates increased abundance in iNPH relative to CO; −, decreased abundance in iNPH relative to CO; CO indicates control; iNPH, idiopathic normal pressure hydrocephalus; log2 Fold Change, effect size measured as fold change and expressed in logarithmic scale (base 2), *P*_adj_, adjusted *P*-value (*P*≤0.05).

**FIGURE 1:**
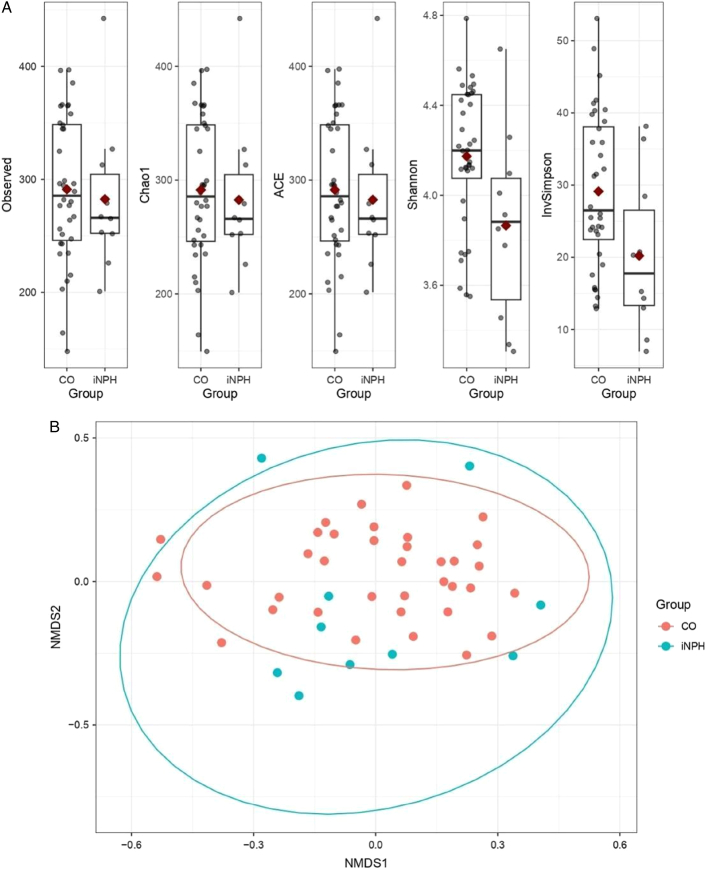
A, Alpha diversity plot by diversity index and group, using ASV-level data. Statistically significant differences in the Shannon and Inverse Simpson indices. B, Beta-diversity NMDS plot based on Bray-Curtis distance. Statistically significant results between the 2 main study groups. ACE indicates an estimated richness index; Chao1, an estimated richness index; InvSimpson, inverse Simpson, richness and evenness index; NMDS, nonmetric multidimensional scaling; Observed, observed richness; Shannon, richness and evenness index.

We did not observe statistically significant alpha diversity differences between the fecal samples from the CO and iNPH groups, in terms of the Chao1, ACE, or Observed Richness indices (Fig. 1). However, the indices describing both the richness and evenness of the samples (the Shannon and inverse Simpson) were significantly lower in the iNPH group, with *P*-values of 0.028 and *P*=0.024, respectively (Fig. 1a). PERMANOVA analysis suggested that the difference between the CO and iNPH samples best explained the beta-diversity (between-sample) microbiome composition variation, with a weak suggestion of potential effects from other clinical factors. Therefore, the finalized beta-diversity (PERMANOVA) model included only the main group variable (CO/iNPH), which showed a *P*-value of 0.0296 in a univariable model, while all other covariates resulted in a *P*-value of >0.1 in the multivariable model (Fig. 1b).

## DISCUSSION

To our knowledge, the present study is the first to investigate the gut microbiome in patients with iNPH, and we identified gut microbiome differences between iNPH patients and healthy participants. It remains unknown, whether the gut microbiome changes precede the pathogenesis of iNPH or if the changes are a result of brain pathology, as this has not been studied before. However, in AD and PD, there are some preliminary findings suggesting that dysbiosis might precede neuropathology.^7,10^

We designed this study to examine possible changes in the microbiome in patients with iNPH, based on increasing evidence in the literature linking the gut microbiome to other neurological diseases, such as Parkinson and Alzheimer diseases.^7,10^ The iNPH patients showed signs of cognitive impairment, as previously reported by Nerg et al.^27^ In contrast, the iNPH and CO groups did not demographically differ from each other in terms of age, sex, or education.

### Differential Abundance Analysis

Our present results revealed ten bacterial genera that differed in abundance between healthy controls and patients with iNPH. The abundances of *Escherichia/Shigella*, and *Anaeromassilibacillus* were clearly increased in the iNPH group compared with the CO group. The iNPH group also showed significantly greater abundances of *Butyrivibrio*, *Duncaniella*, and an unidentified genus from the class Alphaproteobacteria. In addition, compared with the CO group, iNPH patients exhibited deceased abundances of *Agathobaculum*, *Paramuribaculum*, *Catenibacterium*, an unidentified genus from the family Muribaculaceae, and an unidentified genus from the order Bacteroidales.

*Anaeromassilibacillus* is a relatively new genus described in 2017, and it belongs to the Clostridiaceae family, which shows correlations with a variety of diseases. *Anaeromassilibacillus* itself is reportedly correlated with social anxiety disorder.^28^ A murine study demonstrated that gut dysbiosis enabled *Anaeromassilibacillus* and lipopolysaccharides (LPS) to translocate outside of the gut. Gut dysbiosis disrupts the intestinal epithelial barrier, which can lead to intestinal inflammation, increased epithelial permeability, and, finally, bacterial translocation.^29^ Notably, bacterial translocation can promote systemic inflammation and activate the immune system.^30^

*Escherichia/Shigella* (ES) is a well-known pathogenic bacterial genus that reportedly promotes inflammation through the production of harmful Shiga toxins and LPS and by reducing the beneficial genera that produce anti-inflammatory short chain fatty acids (SCFA) in the gut.^31^ The role of Shiga toxins and LPS in neurodegeneration has been demonstrated in a murine model. The combination of these compounds damaged astrocytes and caused striatal microvascular damage, which led to increased blood-brain barrier permeability, and these changes correlated with an increased risk of motor deficits and neurovascular damage.^32^ Both compounds are also reportedly correlated with increased permeability of the intestinal epithelial layer, which enables bacteria to migrate from the lumen and activate a systemic immune reaction.^30^ ES bacteria can also adhere to the intestinal epithelium, and the genus is positively correlated with proinflammatory cytokines that negatively affect the intestinal barrier.^33^

ES has also been reported to be increased in patients with PD,^5^ and increased in blood and fecal samples from patients with AD and mild cognitive impairment.^34^ Moreover, ES is increased in amyloid-positive patients, and gut microbiome differences have been correlated with systemic inflammation.^35^ ES is also reportedly correlated with other diseases, including amyotrophic lateral sclerosis, bipolar disorder, multiple sclerosis, generalized anxiety disorder, and schizophrenia.^31^ Moreover, ES is not exclusively correlated with brain disorders but also with other diseases involving immunologic pathways, for example, rheumatoid arthritis.^36^

### Alpha and Beta Diversity

Regarding alpha diversity, a sample is considered more diverse when it comprises a larger number of different taxa.^24^ The Chao1, Observed Richness, and ACE indices only evaluate the richness of taxa in a sample and do not provide as much information about the actual sample structure compared with the inverse Simpson and Shannon indices, which reflect both richness and evenness. Therefore, it was more informative to use the inverse Simpson and Shannon indices, which revealed differences in alpha diversity between the 2 groups. Specifically, the iNPH group exhibited significant decreases in both inverse Simpson and Shannon, suggesting that alpha diversity was lower in the iNPH group relative to the CO group (Fig. 1A). Notably, alpha diversity is not a biomarker in PD.^37^

Beta diversity differed between the CO and iNPH groups, suggesting that the overall community composition was significantly affected in patients with iNPH (Fig. 1B). Due to a variety of factors, diet strongly affects the gut microbiome composition in numerous ways.^38^ However, our study subjects all came from the same region in Eastern Finland, which suggests that the data were not strongly affected by diet, and that effect was due to the medical condition when measured in terms of bulk bacterial community composition.

### Strengths and Limitations

The sample size of this study was relatively small, which does not provide adequate statistical power to draw bold conclusions. However, we were able to detect differences between groups at several levels. This study in itself showed that iNPH was also associated with changes in the gut microbiome, as has been found for other neurodegenerative disorders.

Among the strengths of this study was the successful formation of the iNPH group, consisting only of pure iNPH cases who did not have other types of dementia, such as AD or vascular dementia. This enabled us to study exclusively the connection between gut microbiome and iNPH without mixing factors. One way to ensure that the iNPH participants were pure cases was to use APOE4-status as an excluding criteria since it is not a risk factor for iNPH. Strict exclusion criteria might also be seen as a limiting factor. It is likely that in mixed-type dementia, more changes could have been found between groups.

### Implications and Future Studies

Since iNPH is a progressive neurodegenerative disorder, early diagnosis is important to maintain patients’ quality of life.^39,40^ It is also crucial to start treatment as early as possible. Gut microbiome biomarkers would enable easy and noninvasive differential diagnostic testing from fecal samples. In the future, it would be interesting to study a larger cohort of patients and to broaden this investigation. In particular, it would be intriguing to examine whether shunting leads to changes in the microbiome and whether the microbiome affects the treatment response among shunted patients.

The genera presented in this report are newly discovered in iNPH. Thus, there is a need for further studies to determine their potential clinical relevance in iNPH and in human health and disease.

## CONCLUSIONS

Here we provide the first demonstration that iNPH patients exhibited different abundances of 10 bacterial genera, as well as differences in alpha diversity and beta diversity, compared with healthy control participants. Notably, 2 of these genera have been previously reported to be associated with increasing bacterial transportation from the gut to the circulatory system, causing inflammation. These changes in bacterial genera abundance are likely not exclusive to neurodegenerative disorders but also found in other neurological diseases and disease processes with an immunologic pathogenesis. These results encourage further studies to be performed in the medical field.
